# Notch ratio in pulmonary flow predicts long-term survival after pulmonary endarterectomy for chronic thromboembolic pulmonary hypertension

**DOI:** 10.1007/s00380-024-02422-5

**Published:** 2024-06-05

**Authors:** M. A. M. Beijk, J. A. Winkelman, H. M. Eckmann, D. A. Samson, A. P. Widyanti, J. Vleugels, D. C. M. Bombeld, C. G. C. M. Meijer, H. J. Bogaard, Anton Vonk Noordegraaf, H. A. C. M. de Bruin-Bon, B. J. Bouma

**Affiliations:** 1 Department of Cardiology, Heart Center, Amsterdam UMC, Amsterdam Cardiovascular Sciences, Room B2-250, Meibergdreef 9, 1105 AZ Amsterdam, The Netherlands; 2 Department of Cardiothoracic Surgery, Heart Center, Amsterdam UMC, Amsterdam Cardiovascular Sciences, Amsterdam, The Netherlands; 3 Department of Pulmonary Medicine, Amsterdam UMC, Amsterdam Cardiovascular Sciences, Amsterdam, The Netherlands

**Keywords:** Chronic thromboembolic pulmonary hypertension, Pulmonary endarterectomy, Notch ratio, Echocardiography, Long-term outcomes

## Abstract

**Background:**

Assessment of the pattern of the RV outflow tract Doppler provides insights into the hemodynamics of chronic thromboembolic pulmonary hypertension (CTEPH). We studied whether pre-operative assessment of timing of the pulmonary flow systolic notch by Doppler echocardiography is associated with long-term survival after pulmonary endarterectomy (PEA) for CTEPH.

**Methods:**

Fifty-nine out of 61 consecutive CETPH patients (mean age 53 ± 14 years, 34% male) whom underwent PEA between June 2002 and June 2005 were studied. Clinical, echocardiographic and hemodynamic variables were assessed pre-operatively and repeat echocardiography was performed 3 months after PEA. Notch ratio (NR) was assessed with pulsed Doppler and calculated as the time from onset of pulmonary flow until notch divided by the time from notch until end of pulmonary flow. Long-term follow-up was obtained between May 2021 and February 2022.

**Results:**

Pre-operative mean pulmonary artery pressure (mPAP) was 45 ± 15 mmHg and pulmonary vascular resistance (PVR) was 646 ± 454 dynes.s.cm-5. Echocardiography before PEA showed that 7 patients had no notch, 33 had a NR < 1.0 and 19 had a NR > 1.0. Three months after PEA, echocardiography revealed a significant decrease in sPAP in long-term survivors with a NR < 1.0 and a NR > 1.0, while a significant increase in TAPSE/sPAP was only observed in the NR < 1.0 group. Mean long-term clinical follow-up was 14 ± 6 years. NR was significantly different between survivors and non-survivors (0.73 ± 0.25 vs. 1.1 ± 0.44, p < 0.001) but no significant differences were observed in mPAP or PVR. Long-term survival at 14 years was significantly better in patients with a NR < 1.0 compared to patients with a NR > 1.0 (83% vs. 37%, *p* =  < 0.001).

**Conclusion:**

Pre-operative assessment of NR is a predictor of long-term survival in CTEPH patients undergoing PEA, with low mortality risk in patients with NR < 1.0. Long-term survivors with a NR < 1.0 and NR > 1.0 had a significant decrease in sPAP after PEA. However, the TAPSE/sPAP only significantly increased in the NR < 1.0 group. In the NR < 1.0 group, the 6-min walk test increased significantly between pre-operative and at 1-year post-operative follow-up. NR is a simple echocardiographic parameter that can be used in clinical decision-making for PEA.

## Introduction

Chronic thromboembolic pulmonary hypertension (CTEPH) is a distinct form of pulmonary hypertension (PH) that is categorized in Group 4 according to the latest clinical classification of PH and current guidelines [1–3]. CTEPH occurs as a result of failure of resolution after deposition of pulmonary thrombo-emboli within the pulmonary circulation and transition of clots into permanent connective tissue [4]. Forming of chronic scar‐like material that partially or completely obstructs the pulmonary vascular bed and vascular remodeling within non-obstructed regions of the pulmonary circulation may lead to an increased pulmonary vascular resistance (PVR) which subsequently leads to progressive PH and right heart failure. Clinical presentation of CTEPH may include progressive exertional dyspnea leading to a decreased exercise capacity, fatigue, and in more advanced stages due to right heart dysfunction, lower extremity edema, elevated jugular venous pressure, ascites and syncope [5]. More than 70% of CTEPH patients have a history of acute pulmonary embolism and the incidence of CTEPH has been reported at 4.8% within 2 years of follow‐up after acute pulmonary embolism [6, 7].

The diagnosis of CTEPH is based on the presence of the following hemodynamic and radiological findings after at least 3 months of appropriate anticoagulation treatment: (a) precapillary PH at right heart catheterization (RHC), defined as an elevated resting mean pulmonary artery pressure (mPAP) of > 20 mmHg and a pulmonary artery wedge pressure < 15mmHg, (b) mismatched perfusion defects on ventilation/perfusion lung scan, and c) specific imaging evidence and vascular changes due to chronic clot material seen by multidetector computed tomography angiography, magnetic resonance imaging, or conventional pulmonary angiography (e.g. eccentric or mural lining thrombus, ring‐like stenoses, webs/slits, and chronic total occlusions) [2, 8].

The prognosis of CTEPH without intervention is poor and proportional to the hemodynamic severity of PH [9]. Assessment of the dominant lesion’s location, i.e. central or peripheral CTEPH, is important to establish treatment options. Besides medical therapy, the treatment of choice, in the presence of anatomically (central) suitable disease and appropriate surgical candidacy, is pulmonary endarterectomy (PEA) [10]. PEA has been shown to improve patients’ hemodynamic (including a marked decrease in mPAP and PVR resulting in an improved right heart function), functional status and exercise capacity when compared to medical treatment sustained throughout long‐term follow‐up [11–13]. At experienced centers, the in‐hospital mortality rate following PEA is relatively low and is generally expected to remain < 5% [12, 14]. Survival rates at 5 and 10 years have been reported to be 84%–93% and 83%, respectively [12].

However, not all patients undergoing PEA may have a favorable outcome and identification of these patients is challenging. Studies have shown that presence of a PVR > 1000 dynes.s.cm^−5^ and/or mPAP > 50 mmHg has a higher likelihood of peri-operative mortality [15–17]. In addition, we have shown that in patients with CTEPH undergoing PEA, a notch ratio (NR) > 1 as assessed by Doppler echocardiography is associated with in-hospital mortality and residual PH (sPAP > 40 mmHg) [18]. NR is the timing of the pulmonary flow systolic notch (mid-systolic deceleration in pulmonary flow) [19]. Whether this simple echocardiographic parameter is associated with long-term outcome is currently unknown. The purpose of this study was to evaluate whether pulmonary flow systolic NR is associated with long-term survival.

## Materials and methods

The design of this study has been reported previously [18]. In brief, in this single-center, non-randomized, prospective study, CTEPH patients who were deemed eligible for PEA after pre-operative assessment were recruited for this study between June 2002 and July 2005. After they gave written informed consent, they were included in this study. There were no exclusion criteria. All investigations were approved by the local institutional review board. Written informed consent was obtained from each patient. Pre-operative assessment included transthoracic echocardiography, pulmonary angiography, and determination of plasma levels of brain natriuretic peptide (BNP). Forty-six patients performed 6-min walk test. A RHC was performed using a Swan-Ganz catheter to measure mPAP, pulmonary capillary wedge pressure (PCWP), cardiac output (CO), and right atrial pressure (RAP), and to be able to calculate PVR and cardiac index.

Transthoracic echocardiographic images (M-mode, two-dimensional, and Doppler) were obtained with a 1.6–3.2 MHz transducer (System Seven, General Electric, USA), digitized, and analyzed offline. From the apical four-chamber view, right ventricular end-diastolic diameter (RVEDD), tricuspid annular plane systolic excursion (TAPSE), and tricuspid regurgitation (TR) jet were recorded. The velocity of the TR jet was used to obtain pulmonary artery systolic pressure (sPAP) from the calculated right ventricle-to-right atrium systolic pressure gradient (Bernoulli equation), to obtain sPAP, RAP values were added to the calculated gradient, with RAP estimated using the collapsibility index of the inferior caval vein in each patient. The severity of TR was quantified according to recommendations of American Society of Echocardiography’s Nomenclature and Standards Committee and The Task Force on Valvular Regurgitation [20]. From the parasternal short-axis view, pulmonary artery systolic flow was recorded by placing the pulsed Doppler sample volume in the middle of the right ventricular outflow tract just proximal to the pulmonic valve orifice.

**Fig. 1:**
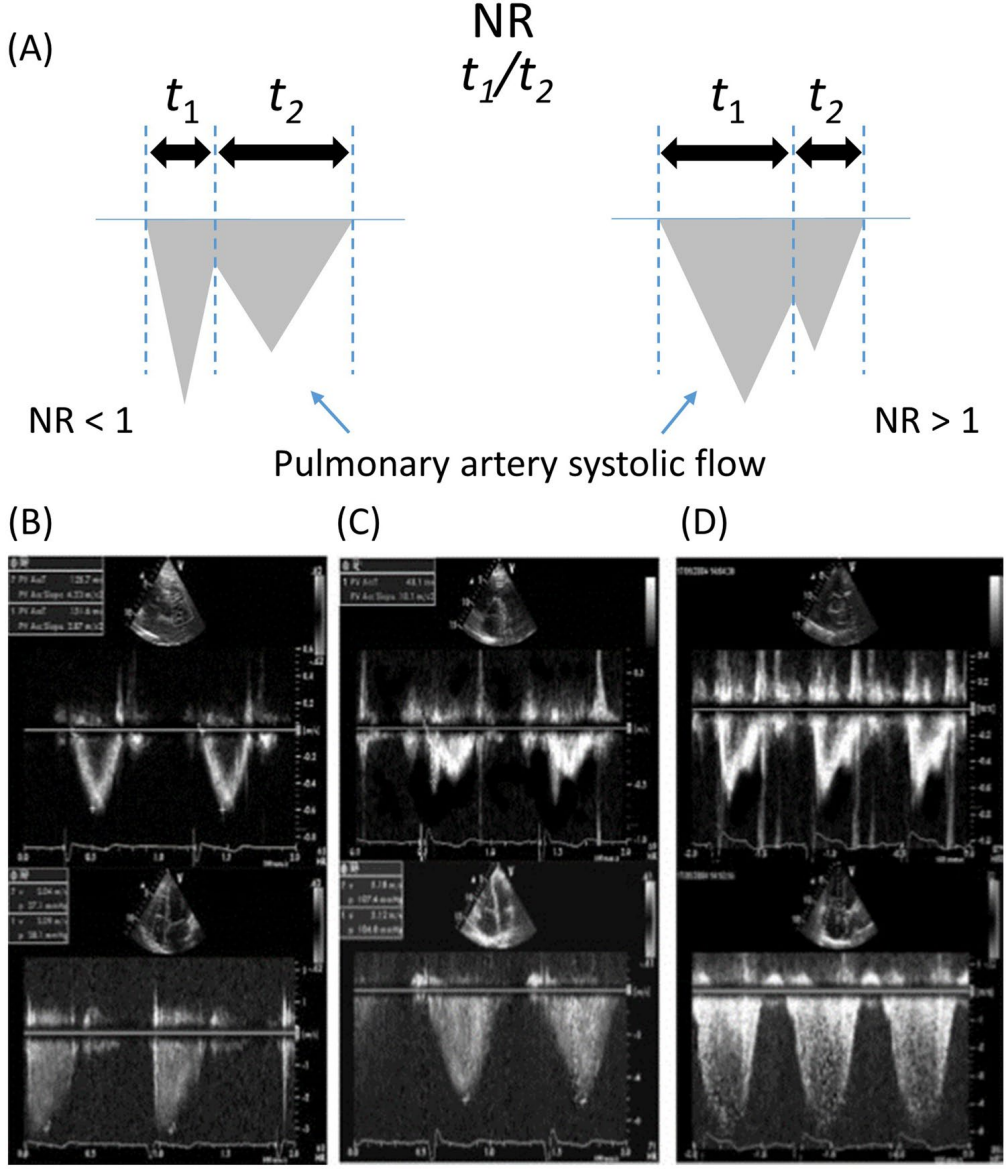
Schematic illustration of the method to calculate pulmonary flow systolic NR. **A** The time interval from the onset of pulmonary artery systolic flow to the maximal systolic flow deceleration (t1) was divided by the time interval from the maximal systolic flow deceleration to the end of pulmonary artery systolic flow (t2). **B**, top Pulmonary flow without notch of a patient with exercise-induced pulmonary hypertension (parasternal short-axis view). (Bottom) Maximal tricuspid regurgitation flow (four-chamber view), used to calculate systolic pulmonary artery pressure. (C, top) Pulmonary flow systolic notch, NR < 1.0. (Bottom) Maximal tricuspid regurgitation flow. (D, top) Pulmonary flow systolic notch, NR > 1.0. (Bottom) Maximal tricuspid regurgitation flow. Note that timing of notch differs between panels (**C** and **D**), despite similar amplitudes of tricuspid regurgitation flow. All demonstrated recordings of pulmonary flow (**B**–**D**) were performed at rest

The pulmonary flow systolic NR was averaged from 2 to 4 consecutive heart beats and was calculated as depicted in Fig. 1. The time interval from the onset of pulmonary artery systolic flow to the maximal systolic flow deceleration (t1) was divided by the time interval from the maximal systolic flow deceleration to the end of pulmonary artery systolic flow (t2).

### Surgical methods

PEA surgery was performed via median sternotomy and with cardiopulmonary bypass. Deep hypothermia circulatory arrest was applied but limited to 20-min intervals. The procedure was bilateral, and on completion of the right pulmonary artery, bypass was resumed and the patient reperfused while the arteriotomy is closed so that the procedure can be repeated on the left side, with circulatory arrest being initiated as necessary [21]. The surgical findings confirmed the diagnosis of CTEPH in all patients included in this study. All patients were operated on by 1 surgeon and concomitant persistent foramen ovale closure was performed in 14 patients.

### Follow-up

Post-operative transthoracic echocardiographic assessment was performed 3 months after PEA. After vital status was checked, clinical follow-up by telephone was performed between May 2021 and February 2022.

### Statistical analysis

Categorical variables are presented as number and percentages and were compared between groups, using a Fisher’s exact test. Continuous variables were checked for normal distribution, using the Wilk–Shapiro test, and presented as mean ± SD in the case of a normal distribution and median and interquartile range (IQR) otherwise. Baseline values of survivors and non-survivors were compared using a two-tailed unpaired Student’s t test or Mann–Whitney test where appropriate. To identify which pre-operative variables were associated with mortality, all variables were included in a univariate logistic binary regression analysis. Variables with a *P* value < 0.10 were entered into a multivariable model. Parameters between patients with no notch, NR < 1.0, and NR > 1.0 pre-operatively and post-operatively were compared using ANOVA. Time-to-event data are reported and displayed as a Kaplan–Meier estimate for event-free survival with comparison between groups by the log-rank test. Finally, a landmark analysis was performed dividing the entire follow-up into initial 1 year and the following period. All analyses were performed using SPSS version 28 (IBM Corp., Armonk, NY, USA), and a *p* value < 0.05 was considered statistically significant.

## Results

From the 61 consecutive CTEPH patients whom underwent PEA, 2 patients were excluded from analysis as the pre-operative echocardiographic variables were incomplete. Thus, a total of 59 patients were included in this retrospective study.

The baseline characteristics of the study population are summarized in Table 1. Overall, mean age was 53 ± 14 years, 34% were male patients and 66% of patients were New York Heart Association (NYHA) functional class III or IV. Pre-operative mPAP was 45 ± 15 mmHg and PVR was 646 ± 454 dynes.s.cm^−5^. Pre-operatively, 7 patients had no notch, 33 had NR < 1.0 and 19 had NR > 1. At the time of pre-operative assessments, 5 patients were receiving bosentan (4 had a notch ratio (NR) < 1.0 and 1 had NR > 1.0), 1 patient received sildenafil (NR < 1.0) and 2 patients were treated with intravenous epoprostenol (1 had NR < 1.0, the other NR > 1.0). Mean long-term follow-up was 14 ± 6 years. Overall, 6 patients died in-hospital (persistent pulmonary hypertension (*n* = 1), intractable bleeding (*n* = 2), persistent pulmonary hypertension resulting in right ventricular failure (*n* = 3)). All patients who died in-hospital had NR > 1.0. Overall, 53 (90%) patients were discharged alive of whom an additional 13 patients died during follow-up, 1 (14%) patient with no notch, 6 patients (18%) with NR < 1.0, and 6 patients (32%) with NR > 1.0. Between survivors and non- survivors, no significant differences were observed in baseline 6-min walking distance (409 ± 114 m. vs. 364 ± 110 m., *p* = 0.22), plasma BNP (9.3 ± 42.6 pmol/l vs. 50.8 ± 77.3 pmol/l, *p* = 0.10), mPAP (43 ± 14 mmHg vs. 49 ± 15 mmHg, *p* = 0.14), PVR (572 ± 431 dynes.s.cm-5 vs. 785 ± 475 dynes.s.cm-5, *p* = 0.098) (Table 1). NR was significant different between survivors and non-survivors (0.73 ± 0.25 vs. 1.1 ± 0.44, *p* < 0.001).

**Table 1:** Baseline patient characteristics

	All patients (*n* = *59*)		Survivors (*n* = *40*)		Non-survivors (*n* = *19*)		*p* value*
Age, years^a^	53	± 14	49	± 13	62	± 12	< 0.001
Male, *n (%)*	20	(34)	10	(25)	10	(53)	0.036
CTEPH duration, years^b^	2.0	(4.0)	2.0	(3.8)	2.0	(3.0)	0.70
NYHA functional class, *n (%)*							0.079
II	20	(34)	17	(43)	3	(16)	
III	36	(61)	22	(55)	14	(74)	
IV	3	(5,1)	1	(3)	2	(11)	
Plasma BNP, pmol/L^b^	12.7	± 59.7	9.2	± 42.6	50.8	± 77.3	0.10
6-MWT, m^a^	395	± 114	409	± 114	364	± 110	0.22
Right heart catheterization							
mPAP, mmHg^a^	45	± 15	43	± 14	49	± 15	0.14
PVR^a^, dyn.s.cm^−5^	646	± 454	572	± 431	785	± 475	0.098
PCWP, mmHg^a^	12	± 6	12	± 6	11	± 6	0.58
RAP, mmHg^a^	10	± 5	10	± 5	11	± 6	0.59
Cardiac output^a^, L/min	4.9	± 2.0	5.1	± 2.2	4.5	± 1.6	0.30
Cardiac index^a^, L/min/m^2^	2.4	± 0.9	2.5	± 0.8	2.1	± 0.9	0.34
Echocardiography							
sPAP, mmHg^a^	74.9	± 24.5	71	± 25	83	± 20	0.087
TAPSE, mm^a^	19	± 5	19	± 5	18	± 5	0.51
TAPSE/sPAP, mm/mmHg^a^	0.30	± 0.18	0.33	± 0.19	0.23	± 0.14	0.71
RVEDD, cm^a^	4.4	± 0.9	4.3	± 1.0	4.4	± 0.9	0.71
PAAT, ms^a^	65	± 19	65	± 18	65	± 21	0.93
Notch absent/notch present	7	/52	6	/40	1	/19	
NR^a^	0.86	± 0.37	0.73	± 0.25	1.1	± 0.44	< 0.001

All variables were obtained at rest. All patients, *n* = 59, except 6-MWT (*n* = 46) and PCWP (*n* = 56). Survivors, *n* = 40, except for 6-MWT (*n* = 31) and PCWP (*n* = 36). Non-survivors, *n* = 19, except for 6-MWT (*n* = 15). *CTEPH* chronic thromboembolic pulmonary hypertension, *NYHA* New York Heart association, *BNP* brain natriuretic peptide, *MWT* minutes walking test, *mPAP* mean pulmonary artery pressure, *TPR* total pulmonary resistance, *PCWP* pulmonary capillary wedge pressure, RAP right atrial pressure, *sPAP* systolic pulmonary artery pressure, *TAPSE* tricuspid annular plane systolic excursion, *RVEDD* right ventricular end-diastolic diameter, *PAAT* pulmonary artery acceleration time, *NR* notch ratio

^*^P-value Survivors vs. Non-survivors

^a^Mean ± SD

^b^Median (IQR)

Table 2 summarizes the pre-operative variables and follow-up of surviving patients with no notch (*n* = 6), early (NR < 1.0, *n* = 27), and late pulmonary flow systolic notch (NR > 1.0, *n* = 7). mPAP and PVR by right heart catheterization and sPAP, TAPSE/sPAP and RVEDD by echocardiography were significant different between no notch and NR < 1.0 or NR > 1.0. In the surviving patients NR was significantly different between NR < 1.0 and NR > 1.0 (0.63 ± 0.16 vs. 1.12 ± 0.08, p < 0.001). At 1-year follow-up, the 6-min walk test in the NR < 1.0 group was significantly better than in the NR > 1.0 group. Moreover, only in the NR < 1.0 group a significant improvement was observed between pre-operative and 1-year post-operative 6MWT (Table 3).

**Table 2:** Pre-operative variables and follow-up of surviving patients with no, early, and late pulmonary flow systolic notch (*n* = 40)

	No notch (*n* = *6*)		NR < 1.0 (*n* = *27*)		NR > 1.0 (*n* = *7*)		*p* value
Age, years^a^	43	± 5	49	± 14	53	± 14	0.41
Male, *n (%)*	0	-	10	(37)^c^	0	-	0.04
CTEPH duration, years^b^	1.5	(1.1)	3.0	(4.5)	2.0	(2.0)	0.15
NYHA functional class, *n (%)*							0.26
II	5	(83)	9	(33)	3	(43)	
III	1	(16)	17	(63)	4	(57)	
IV	0	-	1	(4)	0	-	
Plasma BNP, pmol/L^b^	5.6	± 7.8	12.7	± 68.3	9.2	± 84.3	0.29
6-MWT, m^a^	459	± 43	417	± 119	335	± 110	0.23
Right heart catheterization							
mPAP, mmHg^a^	22	± 7	47	± 12^c^	47	± 15^c^	< 0.001
PVR^a^, dyn.s.cm^−5^	174	± 72	670	± 448^c^	560	± 345^c^	0.036
PCWP, mmHg^a^	8	± 4	13	± 6	14	± 6	0.21
RAP, mmHg^a^	6	± 3	10	± 5	12	± 5	0.058
Cardiac output^a^, L/min	6.3	± 1.3	5.0	± 2.4	4.5	± 0.8	0.34
Cardiac index^a^, L/min/m^2^	2.9	± 0.2	2.5	± 1.2	2.3	± 0.4	0.64
Echocardiography							
sPAP, mmHg^a^	37	± 11	78	± 21^c^	76	± 28^c^	< 0.001
TAPSE, mm^a^	20	± 3	19	± 5	19	± 6	0.84
TAPSE/sPAP, mm/mmHg^a^	0.52	± 0.13	0.27	± 0.13^c^	0.31	± 0.19^c^	0.014
RVEDD, cm^a^	3.4	± 0.2	4.6	± 1.0^c^	4.1	± 0.7^c^	< 0.001
PAAT, ms^a^	95	± 20	60	± 11	59	± 19	0.45
NR^a^	-		0.63	± 0.16	1.12	± 0.08^d^	< 0.001
Follow-up							
6-MWT, m^a^ at 1-year follow-up	477	± 18	534	± 89*	443	± 51^d^	0.04
Long-term clinical follow-up							
NYHA class							0.28
I	4	(80%)	20	(80%)	3	(50%)	
II	1	(20%)	4	(16%)	2	(33%)	
III	0	-	1	(4%)	1	(17%)	
Medication use							
Coumarins	6	(100%)	27	(100%)	7	(100%)	-
Diuretics	0	-	3	(12%)	2	(33%)	0.20
PH medication	0	-	2	(7%)	1	(14%)	0.43

All variables were obtained at rest. No notch, *n* = 6, except for 6-MWT (*n* = 4). NR < 1.0, *n* = 27, except for 6-MWT (*n* = 22) and PCWP (*n* = 25). NR > 1.0, *n* = 7, except for 6-MWT (*n* = 5) and PCWP (*n* = 6). *NR* notch ratio, *CTEPH* chronic thromboembolic pulmonary hypertension, *NYHA* New York Heart association, *BNP* brain natriuretic peptide, *MWT* minutes walking test, *mPAP* mean pulmonary artery pressure, *TPR* total pulmonary resistance, *PCWP* pulmonary capillary wedge pressure, *RAP* right atrial pressure, *sPAP* systolic pulmonary artery pressure, *TAPSE* tricuspid annular plane systolic excursion, *RVEDD* right ventricular end-diastolic diameter, *PAAT* pulmonary artery acceleration time, *PH* pulmonary hypertension

^a^Mean ± SD

^b^Median (IQR)

^c^*p* < 0.05 vs. No notch

^d^*p* < 0.05 vs. NR < 1.0

^*^p < 0.05 post-operative vs. pre-operative within group

**Table 3:** Three months post-operative echocardiographic follow-up of patients alive at discharge (*n* = 53)

	NR < 1.0					NR > 1.0				
	Survivors (*n* = *27*)		Non-survivors (*n* = *6*)		*p* value	Survivors (*n* = *7*)		Non-survivors (*n* = *6*)		*p* value
sPAP, mmHg^a^	33	± 10*	51	± 32	0.015	42	± 14*	61	± 30	0.16
TAPSE, mm^a^	16	± 3*	16	± 3*	0.84	16	± 3	15	± 2	0.47
TAPSE/sPAP, mm/mmHg^a^	0.55	± 0.21*	0.47	± 0.24	0.45	0.43	± 0.17	0.26	± 0.12	0.10
RVEDD, cm^a^	3.4	± 0.63*	3.4	± 0.46*	0.83	3.7	± 0.81*	4.1	± 0.55	0.45
PAAT, ms^a^	95	± 20*	74	± 28	0.064	89	± 16*	92	± 37	0.87

*NR* notch ratio, *sPAP* systolic pulmonary artery pressure, *TAPSE* tricuspid annular plane systolic excursion, *RVEDD* right ventricular end-diastolic diameter, *PAAT* pulmonary artery acceleration time

^a^Mean ± SD

^*^p < 0.05 post-operative vs. pre-operative within group

**Fig. 2:**
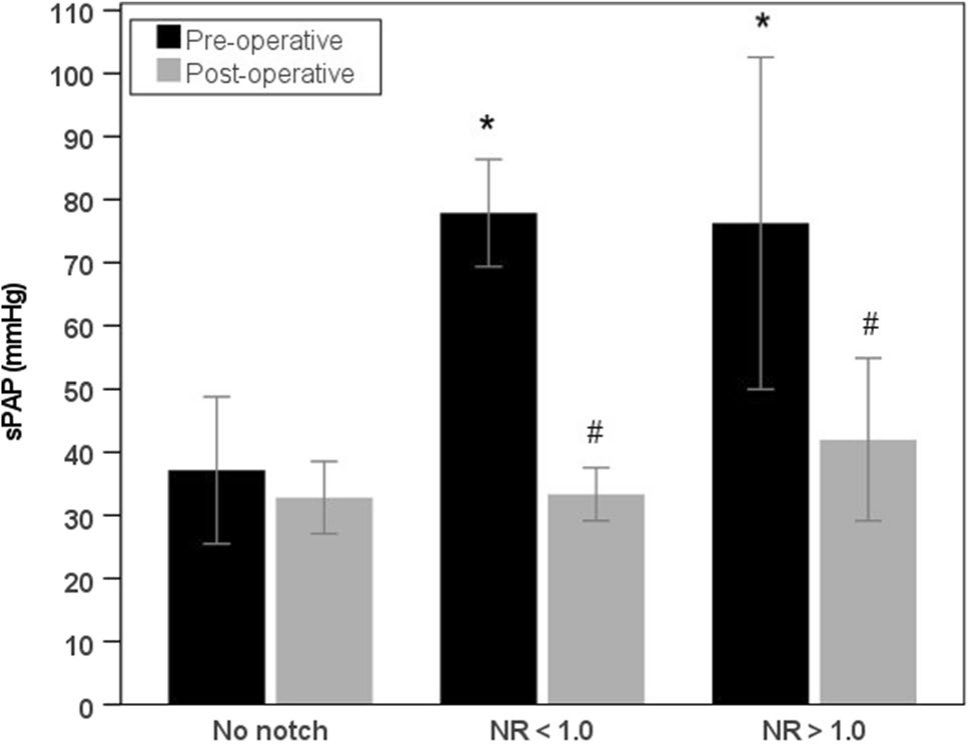
Pre-operative and post-operative sPAP. Pre-operative and post-operative (3 months after PEA) sPAP of surviving patients with no pulmonary flow systolic notch, NR < 1.0 and NR > 1.0; *P < 0.05 vs. no notch (pre-operative); #P < 0.05 post-operative vs. pre-operative within group

**Fig. 3:**
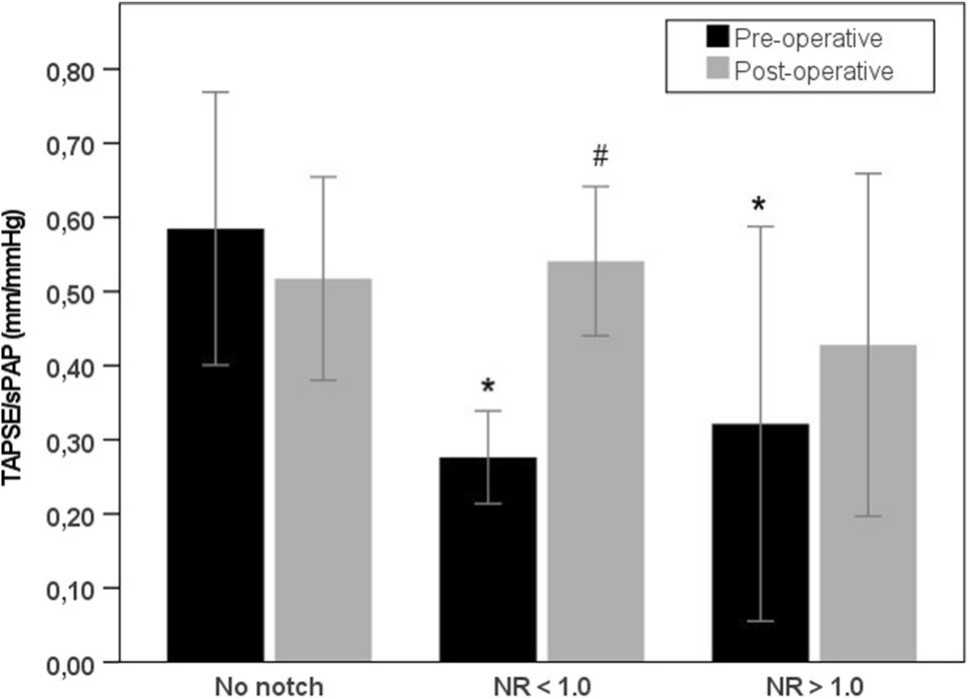
Pre-operative and post-operative TAPSE/sPAP. Pre-operative and post-operative (3 months after PEA) TAPSE/sPAP of surviving patients with no pulmonary flow systolic notch, NR < 1.0 and NR > 1.0; *P < 0.05 vs. no notch (pre-operative); ^#^*P* < 0.05 post-operative vs. pre-operative within group

Three-month post-operative echocardiographic follow-up of patients alive at discharge (*n* = 53) are presented in Table 3. In patients with NR < 1.0, a significant difference was only observed in sPAP between long-term survivors and non-survivors (33 ± 10 mmHg vs. 51 ± 32 mmHg, *p* = 0.015). In patients with NR > 1.0, no significant differences were observed between long-term survivors and non-survivors. No significant differences were observed between echocardiographic parameters between patients with no notch (data not shown), NR > 1.0 or NR < 1.0. To illustrate the utility of NR = 1.0 as cut-off value to predict hemodynamic improvement, we compared pre-operative and 3-month post-operative echocardiographic parameters between patients with no notch, NR < 1.0 and those with NR > 1.0. In the no notch group, there were no differences in pre-operative and post-operative echocardiographic findings (data not shown). In the NR < 1.0 group, significant changes were observed between pre-operative and post-operative echocardiographic sPAP (for long-term survivors only) (Fig. 2), TAPSE (for long-term survivors and non-survivors), TAPSE/sPAP (for long-term survivors only) (Fig. 3), RVEDD (for long-term survivors and non-survivors) and pulmonary artery acceleration time (PAAT) (for long-term survivors only). In the NR > 1.0, significant changes were between pre-operative and post-operative echocardiographic sPAP (Fig. 2), RVEDD and PAAT (for long-term survivors only) but not in TAPSE or TAPSE/sPAP (Fig. 3 and Table 3).

**Fig. 4:**
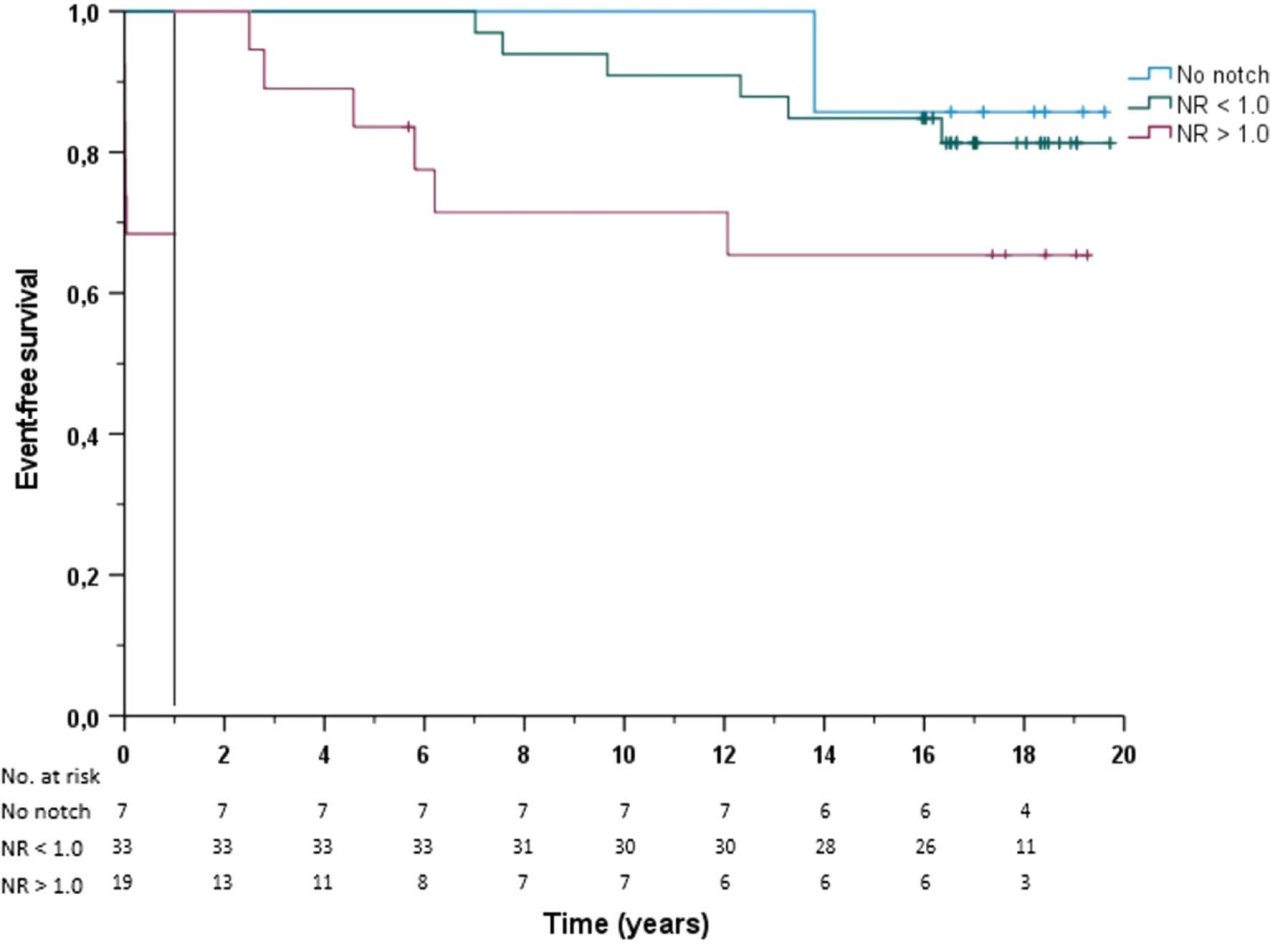
Kaplan–Meier event-free survival curves based on the notch ratio. Event-free survival is subdivided into the first year and thereafter for patients with no notch (blue line), NR < 1.0 (green line), and NR > 1.0 (red line)

In a logistic binary regression analysis of the pre-operative determinants of mortality after PEA together with age, NR was the strongest predictor for long-term mortality (Table 4). Overall long-term survival at 1, 5, 8 and 14 years in patients with a NR < 1.0 was significantly better compared to patients with a NR > 1.0 (100%, 100%, 95% and 83% versus 68%, 53%, 42% and 37%, *p* < 0.001). For patients without a notch, survival at 1, 5 and 8 years was 100% and at 14 years 86%. The landmark event-free survival separating early from late mortality is shown in Fig. 4. There was significantly lower rate event-free survival in the NR > 1.0 group beyond 1 year *(P* = 0.022).

**Table 4:** Logistic binary regression analysis of the pre-operative determinants of mortality after PEA

	Univariate analysis		Multivariate analysis	
	OR (95% CI)	*p* value	OR (95% CI)	*p* value
Age	1.09 (1.03—1.15)	0.002	1.09 (1.01—1.17)	0.027
Gender	0.30 (0.10—0.95)	0.04	0.13 (0.02—1.01)	0.51
NYHA	11.3 (0.77—167.97)	0.078	1.83 (0.18—18.72)	0.61
sPAP	1.02 (0.99—1.05)	0.091	1.03 (0.96—1.11)	0.44
TAPSE/sPAP	0.024 (0.001—1.59)	0.081	0.74 (0.0—91,118)	0.96
NR	8.08 (2.34—27.89)	< 0.001	25.65 (3.20—205)	0.002

*NYHA* New York Heart Association, *sPAP* systolic pulmonary artery pressure, *TAPSE* tricuspid annular plane systolic excursion, *NR* notch ratio

## Discussion

The aim of the current study was to evaluate whether pulmonary flow systolic NR, as assessed pre-operative by Doppler echocardiography, was associated with long-term clinical outcomes in CTEPH patients whom underwent PEA. The main findings of this study are: (a) the timing of such a notch within the cardiac cycle is an excellent predictor of long-term survival, with a low risk of mortality in patients with a NR < 1.0; (b) long-term survivors with a NR < 1.0 and NR > 1.0 had a significant decrease in sPAP as assessed by echocardiography 3 months after PEA. However, the TAPSE/sPAP only significantly increased in long-term survivors with a NR < 1.0 group; (c) in the NR < 1.0 group the 6-min walk test increased significantly between pre-operative and at 1-year post-operative follow-up.

The long-term prognosis of patients undergoing PEA was reported in several studies. In a multicenter European registry, a total of 404 patients with CTEPH were prospectively included and estimated survival at 1 and 3 years was 93% and 89% in operated patients and 88% and 70% in not-operated patients [22]. In a recent Chinese single-center study, 76 CTEPH patients were included between 2002 and 2020 and median time of follow-up was 7.29 years [23]. The survival rate at 1, 3, 5, 10, and 15 years postoperatively was 100%, 97%, 95%, 90% and 83%, respectively. These mortality rates are in line with the findings in our study for the patients with NR < 1.0 (100% at 1 year, 100% at 5 years, 95% at 8 years, and 83% at 14 years). Patients with no notch had an even better prognosis after PEA. In contrast, patient with NR > 1.0 had a higher mortality rate (68% at 1 year, 53% at 5 years, 42% at 8 years, and 37% at 14 years) compared to the non-operated patients included in the multi-center study by Delcroix, et al. [22]. Besides the difference in NR, there may have been other confounding factors, cardiac or non-cardiac, contributing to patients' deaths.

According to the recent European Respiratory Society/European Society of Cardiology (ERS/ESC) guidelines [1], PEA remains the treatment of choice in symptomatic patients with CTEPH. Besides the presence of anatomically central thromboembolic lesions suitable for surgery, the patient’s symptoms, comorbidities, the severity of PH, right heart dysfunction, and risk–benefit analysis are all important factors to be able to select appropriate candidates for PEA. The results of our study extend our previous findings that pre-operative NR by echocardiography can be considered as an additional factor in the decision-making to treat patient with PEA. NR is a stronger and more consistent predictor than the more traditional echocardiographic variables, such as mPAP, sPAP, PVR, or indices of right ventricular function, e.g. TAPSE although the mean PVR (646 ± 454 dyn.s.cm^−5^) was not severely increased in our study. In a recent systematic review, the reported values of pre-PEA PVR ranged between 552 and 1536 dyn.s.cm^−5^ [24]. Several studies have shown that high pre-operative PVR values are associated with an increased mortality, with a 3 times higher in-hospital mortality in patients with pre-operative PVR > 1200 dyn.s.cm^−5^ [25, 26]. In our study, pre-operative PVR was not associated with long-term mortality which might be related to the relative low mean PVR in our study population. However, a significant difference was observed between patients with no notch compared to patient with a NR < 1.0 or NR > 1.0. This difference in PVR only translated into a worse prognosis in the NR > 1.0. Importantly, it has been shown that high pre-operative PVR is not necessarily related to worse post-operative outcomes [27]. Therefore, pre-operative PVR value should not be considered alone as an exclusion criterion for PEA. In addition, CTEPH patients with severe elevated mPAP, but NR < 1.0 should be considered eligible for PEA, even if they are severe symptomatic, as their risk of mortality is low. In contrast, CTEPH patients with severe elevated PAP and a NR > 1.0 have a relatively high risk of in-hospital and long-term mortality. Finally, patients with a NR > 1.0 had a less hemodynamic improvement compared to patients with a NR < 1.0. Therefore, pharmacological treatment may be considered in high-risk patients with NR > 1.0.

The ratio of TAPSE and sPAP derived from echocardiography has been validated as a prognostic outcome measurement in PH [28]. Recently Duan, et al. demonstrated that in CTEPH patients, baseline RV-PA coupling measured by echocardiography as the TAPSE/sPAP ratio is associated with disease severity and adverse outcomes [29]. A low TAPSE/sPAP identifies patients with a high risk of clinical deterioration. In our study, TAPSE/sPAP was lower in non-survivors compared to survivors, but this difference did not reach statistical significance (0.23 ± 0.14 vs. 0.33 ± 0.19, *p* = 0.71) which might be explained by a less hemodynamic severity of PH. Noteworthy, of the surviving patients, those with a NR < 1.0 or NR > 1.0 had a significant lower TAPSE/sPAP compared to those without a notch reflecting disease severity. Remarkably, long-term survivors with a NR < 1.0 showed a significant improvement of TAPSE/sPAP ratio at 3 months after PEA while this was not observed in long-term non-survivors or patients with NR > 1.0. Long-term non-survivors with a NR < 1.0 and patients with NR > 1.0 had a limited hemodynamic improvement after PEA which may be explained by distal obstruction of the pulmonary flow caused by distal arteriopathy. Clearly, this deviation cannot be alleviated by surgical intervention. This is supported by the findings of our study that in 4 out of 6 in-hospital deaths (all 6 had a NR > 1.0) were due to persistent PH resulting in RV failure, despite successful surgical removal of thrombo-embolic material. In the two remaining patients (who died intraoperatively from intractable bleeding), post-mortem analysis revealed arteriopathy in the small pre-capillary pulmonary vessels. Noteworthy, although the timing of the pulmonary flow systolic notch is a reoperative determinant of mortality and post-operative hemodynamic improvement, it does not reflect the extent of obstruction of the pulmonary artery vasculature (PAP, PVR), nor the contractility of the left ventricle or RV (RVEDD, RAP), as these measures were similar between patients with NR < 1.0 and those with NR > 1.0. Our findings support the notion that notch timing has a distinct pathophysiological basis.

In CTEPH, pathological alterations in mechanical properties of the pulmonary arteries increase the afterload to the RV. In the early stage of CTEPH, this mild elevation in afterload leads to adaptive RV hypertrophy, allowing for preservation of CO (homeometric adaptation). However, the continuous overloaded RV fails to increase contractility proportionally to the further elevated afterload and eventually uncouples from the pulmonary circulation. It has been shown that RV-pulmonary artery (PA) uncoupling, i.e. a mismatch between RV contractility and its afterload, may serve as an early marker of RV dysfunction [30]. The prognosis of PH largely depends on the preservation of RV function, especially its contractility, to compensate for the afterload. Several mechanical factors seem to affect RV function [31]. The localization of vessel obstruction, either proximal (central) or distal (peripheral), has an impact on RV function in CTEPH [32]. Significant hemodynamic differences have been observed between central and peripheral forms of CTEPH, with worse hemodynamics in the central form of CTEPH which may reflect a different pathophysiological response of the RV between the two forms of CTPEH [32]. Furthermore, additional influences may contribute to this different response especially to peripheral pulmonary bed affection. In patients with proximal CTEPH, a more pronounced RV dilatation and lower RV ejection fraction (RVEF) was observed than in those with distal CTEPH. This difference could not be attributed to well-known lumped arterial parameters of RV load, i.e. PVR and vascular compliance. Wave reflection can provide an additional description of RV load, allowing assessment of mechanical stress (wall stress or tension) as a function of time [33]. RV wall tension in PH is determined not only by pressure, but also by RV volume and a larger volume at a given pressure causes larger RV wall tension. Early return of reflected pressure waves adds RV pressure in early systole, when RV volume is relatively large. In CTEPH patient, it has been shown that the timing of the peak of the reflected pressure wave correlated significantly with RV function, in terms of RV dilatation, RVEF, RV hypertrophy, and RV wall stress [33].

The pulmonary flow systolic notch as assessed by Doppler echocardiography is a non-invasive and easy to perform measurement. The reproducibility of the calculation of NR has been reported previously showing excellent inter-observer and intra-observer correlations (*r* = 0.96 and *r* = 0.98, respectively) [18]. The mean intra-observer difference was 0.06 (range 0.01–0.13) and the mean inter-observer difference was 0.07 (range 0.01–0.18).

The limitations of this study are inherent to the small number of patients. Although this study identifies NR as an independent determinant of PEA outcome, it was limited in number at 59 patients. Seven patients with no notch underwent PEA because of disabling exercise limitation with thrombo-embolic occlusion and exercise-induced PH at right heart catheterization. However, no echocardiographic examination during exercise was performed in these patients.

## Conclusion

Pre-operative assessment of NR is a predictor of long-term survival in CTEPH patients undergoing PEA, with low mortality risk in patients with NR < 1.0. Long-term survivors with a NR < 1.0 and NR > 1.0 had a significant decrease in sPAP after PEA. However, the TAPSE/sPAP only significantly increased in the NR < 1.0 group. In the NR < 1.0 group, the 6-min walk test increased significantly between pre-operative and at 1-year post-operative follow-up. NR is a simple echocardiographic parameter that can be used in clinical decision-making for PEA.

## Data Availability

The data underlying this article will be shared on reasonable request to the corresponding author.
